# First report of Strongylidae nematode from pilot whale (*Globicephala macrorhynchus*) by molecular analysis reveals the cosmopolitan distribution of the taxon

**DOI:** 10.3389/fvets.2023.1313783

**Published:** 2023-12-07

**Authors:** Witchuta Junsiri, Sk Injamamul Islam, Auyarat Thiptara, Autthaporn Jeenpun, Piyanan Sangkhapaitoon, Khunanont Thongcham, Rattanakorn Phakphien, Piyanan Taweethavonsawat

**Affiliations:** ^1^Parasitology Unit, Department of Pathology, Faculty of Veterinary Science, Chulalongkorn University, Bangkok, Thailand; ^2^Epidemiology and Information Group, Veterinary Research and Development Center (Upper Southern Region), Nakhon Sri Thammarat, Thailand; ^3^Animal Diagnostic Group, Veterinary Research and Development Center (Upper Southern Region), Nakhon Sri Thammarat, Thailand; ^4^Marine Endangered Species Unit, Marine and Coastal Resource Research Center, Lower Gulf of Thailand, Department of Marine and Coastal Resources, Thailand; ^5^Biomarkers in Animal Parasitology Research Group, Faculty of Veterinary Science, Chulalongkorn University, Bangkok, Thailand

**Keywords:** ITS2, PCR, short-finned pilot whale, Strongylidae, Thailand

## Abstract

This study investigates the identification, genetic composition, and placement in the evolutionary tree of a particular nematode species found in a short-finned pilot whale in the Gulf of Thailand. To accomplish this, we utilized various methods, including microscopic observations, molecular techniques, and comparative analyses to better understand the characteristics of this parasite. Initially, we concentrated on studying the 18s rDNA sequence through nested PCR, resulting in a 774-bp product. After conducting a BLASTn analysis, we discovered that there were only a few sequences in the GeneBank that shared similarities with our nematode, particularly with *Cyathostomum catinatum*, although the percent identity was relatively low. To confirm the uniqueness of our sequence, we constructed a phylogenetic tree that demonstrated a distinct branch for our nematode, suggesting significant genetic differentiation from *C. catinatum*. Additionally, we sequenced a 399-bp section of the ITS2 gene using PCR, and the resulting data showed a close association with the Strongylidae family, specifically with *Cylicocyclus insigne*. This was further confirmed by BLASTn and CD-HIT-est results, which indicated a 99 and ~94% sequence homology with *C. insigne*, respectively. The ITS2 phylogenetic tree also supported the position of our isolated sequence within the Strongylidae family, clustering closely with *C.insigne*. Our findings shed light on the genetic connections, taxonomy, and evolutionary trends within the Strongylidae family, with a particular focus on the widespread nature of the *Cylicocyclus* genus. This study emphasizes the importance of utilizing molecular techniques and interdisciplinary approaches to gain insight into nematode diversity, evolution, and ecological dynamics in marine environments.

## 1 Introduction

Pilot whales, among cetaceans, are renowned for their sociability and are often found in large groups, occasionally numbering in the hundreds. Their presence in food webs highlights their important role in ecosystem structure and dynamics (1). There are two recognized species of pilot whales: the long-finned pilot whale (*Globicephala melas*), which has gained recognition for its frequent involvement in mass strandings across the globe (2), and the short-finned pilot whale (*Globicephala macrorhynchus*) (3). The short-finned pilot whale is an offshore and deep-diving odontocete cetacean that is found all over the world. It is a member of the Delphinidae family. Short-finned pilot whales can be found in warm temperate seas, which encompass tropical and subtropical regions.

There is a shortage of available information on the parasite fauna found in pilot whales, the incidence of infections, and the accompanying diseases (4). Although no parasites have been reported in the short-finned pilot whale, a small number of bacterial species, such as *Vibrio* and Bacteroides, have been identified (3) and Cetacean morbillivirus (CeMV) (5) have previously been reported. The presence, richness, and nested infra communities within a network of long-finned pilot whale-helminth interactions were examined in a recent study, focusing on the influence of host age and sex (4). Few cases of protozoan *Toxoplasma gondii* infection in cetaceans have been documented in several nations in North America, Western Europe, and Oceania. However, there remains a lack of information regarding this infection in certain regions, including Southeastern Asia, South Asia, South America, and Africa (6). However, a study indicated that there is a plausible chance of toxoplasmosis in cetacean populations in the Philippines by detecting 71% of the sampled cetaceans that were infected with *T. gondii* (7). Recently, a parasite copepod *Pennella balaenoptera*, has only been found as a parasite on marine mammals discovered from a Bryde's whale (*Balaenoptera edeni*; Anderson, 1879) and a pygmy sperm whale (*Kogia breviceps*; Blainville, 1838) on the eastern and southern coast of China in 2022 (8). Moreover, Cetaceans, such as dolphins, function as primary hosts for zoonotic anisakid nematodes, which play a significant role as causative agents for human anisakiasis and health hazards connected with allergies (9). In central Philippine waters within the West Pacific region, *Anisakis typica* was reported from Fraser's dolphin, second only to that reported from the Florida coast, USA isolated from its gut (9, 10). However, despite advancements in marine mammal research, there remains a notable knowledge gap regarding the complex parasite composition and the continuously changing dynamics within the distinct ecosystem of the short-finned pilot whale.

Nematodes have steady abundance and diversity throughout marine, freshwater, and terrestrial habitats, making them the only major metazoan group to possess evolutionary dynamics (11). Strongylid nematodes are often seen as parasites in equine species, including horses, donkeys, mules, and zebras (12). These Strongylids may be classified into two subfamilies, namely the Strongylinae, which encompasses the large strongyles, and the Cyathostominae, which encompasses smaller Strongyles or Cyathostomes (13). Among them, the genus *Cylicocyclus* is recognized as the most widespread taxonomic group within the subfamily Cyathostominae (13). The members of this genus may have a global distribution, inhabiting both terrestrial and marine ecosystems (14, 15). Interestingly, in our study, we reported an equine nematode parasite from the genus *Cylicocyclus* (Family: Strongylidae) from the gastrointestinal tract of the pilot whale by molecular techniques based on the *ITS2* gene for the first time. For the last two decades, scholars have used nucleotide sequences as a means to explore the phylogeny of nematodes (16, 17). In other investigations, there has been an emphasis on obtaining a comprehensive sample of nematodes at the phylum level, which is crucial for the establishment of a comprehensive molecular phylogenetic framework for this group of organisms (18). Researchers also suggested that the integration of the first and second internal transcribed spacer region (ITS-1 + ITS-2 = ITS) inside the nuclear ribosomal DNA (rDNA) has been shown to provide valuable indicators for the discrimination of nematode parasites (19, 20). Thus, this study applied a molecular characterization method to identify isolated nematode parasites from the dead pilot whale based on 18s rDNA and ITS2 gene specifically designed for nematode species (21, 22).

Moreover, this investigation focused on describing a nematode parasite found inside a short-finned pilot whale (*G. macrorhynchus*) that had been bitten by a shark and was discovered off the coast of the Gulf of Thailand. The specimen was taken to a Marine and Coastal Resource Research Center Lower Gulf of Thailand where it underwent pathological examination. The objective of this study was to identify the presence of nematode parasites in the shark-bitten short-finned pilot whale by applying molecular markers. This research adds to our understanding of nematode parasite occurrence among marine mammals found in the region.

## 2 Methods

### 2.1 Host animal and parasite collection

**Figure 1:**
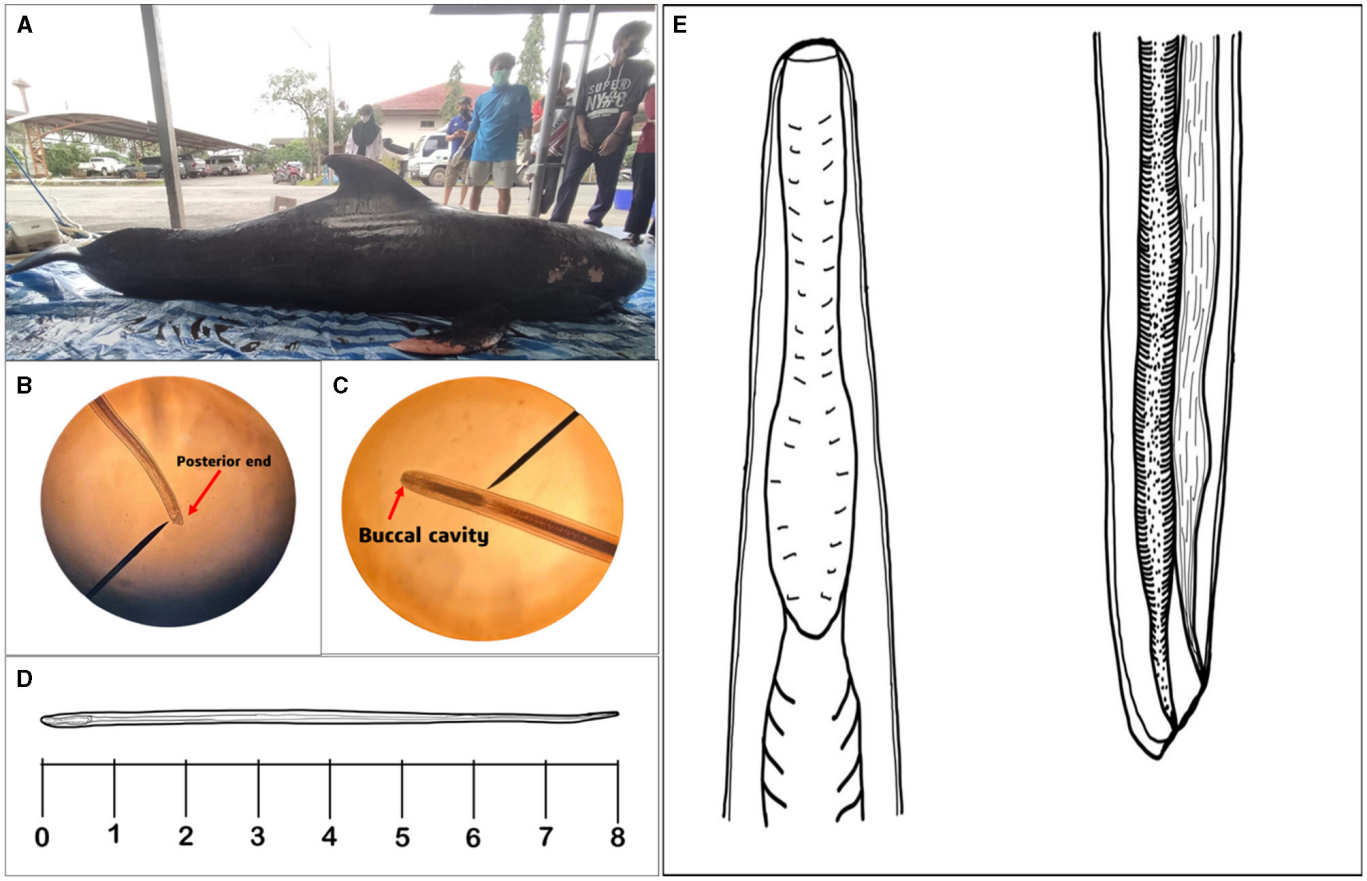
The photographs depict a short-finned pilot whale (*Globicephala macrorhynchus*) and its parasites in precise detail. **(A)** The image shows a deceased short-finned pilot whale. **(B)** The posterior end of the nematode, highlighted by an arrow, indicates that the parasite is female when observed under a light microscope. **(C)** Under light microscopic visualization, the buccal cavity of the parasite can be seen, marked by an arrow. **(D)** Illustration of the full body length of the parasite and **(E)** Present study illustrates the buccal cavity and posterior region of the isolated parasite.

On December 5th, 2022, a 6-year-old male Pilot Whale (*G. macrorhynchus*) was discovered on Narathat Beach in Narathiwat Province, Thailand (6.4477° N and 101.8242° E). Tragically, the whale died on December 7th, 2022 (Figure 1A). The whale's body displayed extensive injuries, including a shark bite, along with concerning clinical findings such as the presence of yellowish bronchi fluid with bronchial bubbles, lung edema with bubbles, yellowish fluid, and clotting blood in the pericardium, clotting blood in the left ventricle, hyperemia in the urinary bladder, as well as edema and clotting blood in the small intestine. Subsequently, during a necropsy examination of the whale, a parasite was recovered from its gastrointestinal tract. The parasite was then preserved in 70% alcohol for further molecular study after an initial microscopic examination.

### 2.2 DNA preparation and molecular identification

Genomic DNA from the sample was extracted using a Nucleospin DNA extraction kit, following the manufacturer's instructions (NucleoSpin^®^, Germany). A modified nested PCR amplification was performed based on the 18S rRNA gene. For the first PCR reaction, two μL of genomic DNA were used in a 20 μL reaction with outer primers G18S4F (GCTTGTCTCAAAGATTAAGCC) at the position 963–983 bp in complete rDNA and 136R (TGATCCTTCTGCAGGTTCACCTAC) at the position 2671–2690 bp (22). For the second PCR, the same 20 μL reaction was prepared with a DNA template from the first PCR using inner primers 18SinF (CAGGTGAGTTTTCCCGTGTT) and 18SinR (AAACGGCTACCACATCCAAG) (23). PCR was done under the same cycle conditions, which included an initial denaturing period at 94°C for 5 min and 35 cycles of 94°C for 30 sec, 55°C for 30 sec, and 72°C for 80 sec, and a final extension for 7 min. Additionally, the *ITS2* gene region was subjected to polymerase chain reaction (PCR) amplification using the conserved 20-mer oligonucleotide primers NC1 and NC2 as described by Gasser et al. (21). Subsequently, the PCR products from both genes were subjected to separation by using a 1.5% agarose gel. The purified expected product obtained from polymerase chain reaction (PCR) was subjected to purification using a commercially available kit, following the procedure provided by the manufacturer (NucleoSpin^®^ Gel and PCR Clean-up, Macherey-Nagel, Düren, Germany). Afterward, a commercial DNA sequencing service sequenced the purified product in both forward and reverse directions (Celemics, Korea).

### 2.3 Sequence analysis and phylogenetic tree construction

The 18S rRNA and ITS2 sequences were subjected to analysis using the NCBI BLASTn program (http://www.ncbi.nlm.nih.gov/BLAST/). The resulting sequences were subsequently deposited in GenBank with the accession number OR478781 from 18s rDNA and OR473063 from the *ITS2* gene. To ascertain the characteristics encoded by the *ITS2* gene, datasets including the ITS2 sequences of each nematode species were compared to the nucleotide sequences of the designated database (https://www.nemabiome.ca/its2-database) (24) using the CD-HIT (Cluster Database at High Identity with Tolerance) Suite: Biological Sequence Clustering and Comparison, specifically focusing on the CD-HIT-est algorithm (25). The cutoff for the minimum acceptable level of sequence identity was set at 80%. The software application accepts a sequence database in fasta format as its input and generates a collection of sample sequences, referred to as “non-redundant” (nr), as its output. Furthermore, the cd-hit-est program generates a cluster file that provides documentation of the sequence “groupies” associated with each sample sequence in the non-redundant (nr) sequence dataset. The objective is to decrease the overall size of the database while retaining all sequence information, achieved by selectively eliminating “redundant” (or very identical) sequences. In essence, the cd-hit-est algorithm generates a collection of nucleotide families that exhibit close relationships based on a provided fasta sequence database. In addition, phylogenetic analysis was performed based on the 18s rDNA and *ITS2* gene dataset after performing BLASTn and retrieving the sequence from the Gene Bank. The details of the sequences are listed in the Supplementary Tables 1, 2. Multiple sequences were aligned using the ClustalW multiple alignments feature of MEGAXv10.2.6 (26). For each dataset (18S rDNA and ITS2), phylogenetic trees were constructed using MEGAX v10.2.6 (27) and visualized by ITOL (https://itol.embl.de/) an interactive web tool for phylogenetic tree visualization. Before constructing the phylogenetic tree, the best model fit for the analysis was determined by using jMODELTEST 2.1.7 (28), which is a tool to carry out a statistical selection of best-fit models of nucleotide substitution; followed by examining using maximum likelihood (ML) methods (29, 30). Potential species were separated using clustering in the ML approach of the optimal nucleotide substitution model, as predicted by the “Kimura 2 parameter model.” It was determined that a gamma distribution of rates and proportion of invariant sites HKY + I + G for 18s rDNA and GTR+G+I for ITS2 would provide the best fit for the ML analysis data (31). Using the bootstrap approach with 1,000 replicates, it was determined that the internal branches in all trees were valid (31).

## 3 Results

### 3.1 Parasite description

The nematode species analyzed in this study exhibit intriguing characteristics when observed under a light microscope, measuring 8 mm in length (Supplementary Figure 1). Figure 1B captures the posterior region of the worm, playing a significant role in determining the nematode's sex. Figure 1C provides a close-up view of the buccal cavity, revealing the specialized feeding apparatus of the nematode. The illustration of the entire body of the nematode is shown in Figure 1D. Lastly, drawing of the parasite enables a more comprehensive examination of the intricate anatomical features found within the parasite's buccal cavity and posterior end (Figure 1E).

### 3.2 Sequence and phylogenetic analysis

**Figure 2:**
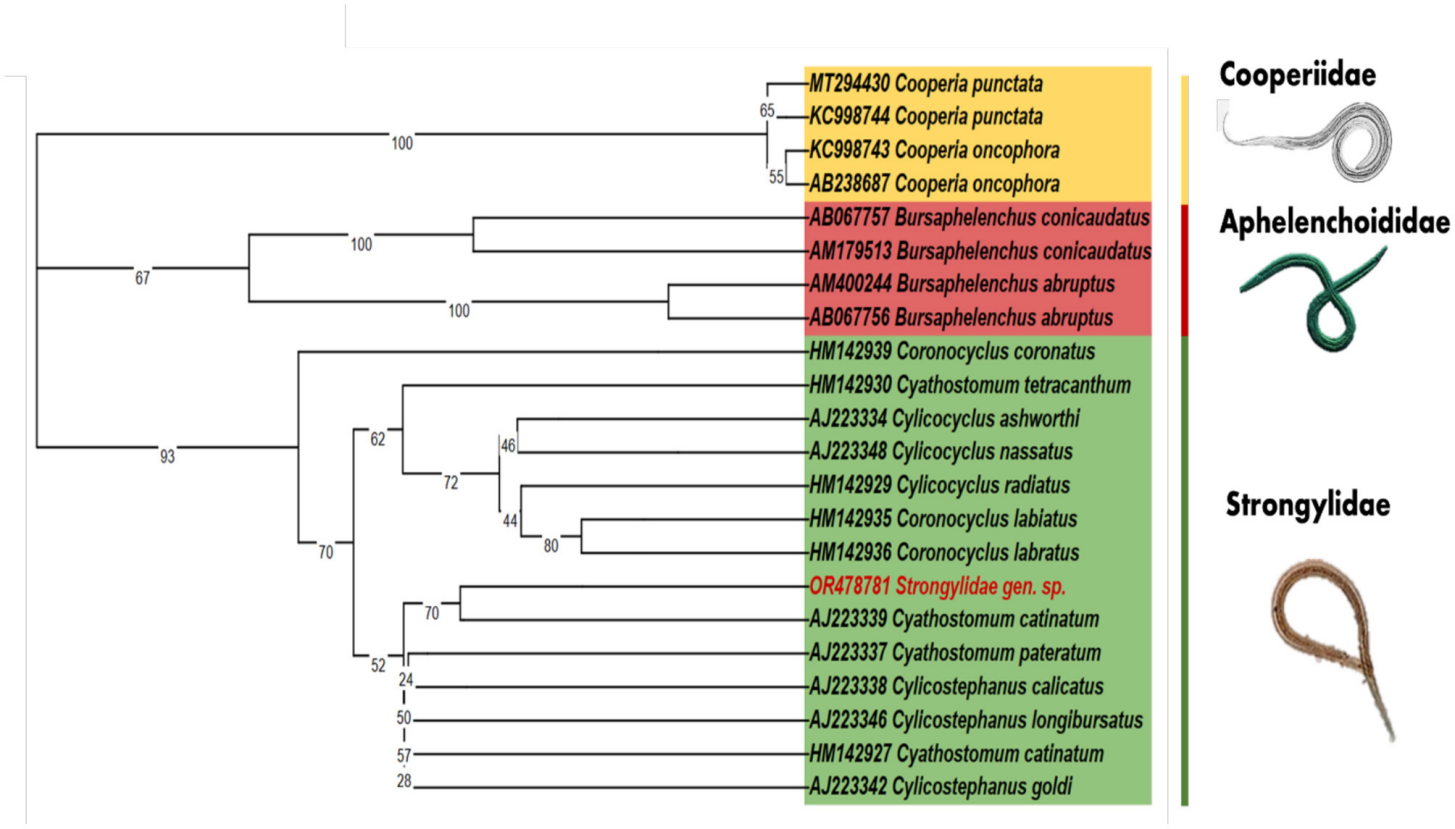
The maximum likelihood analysis of the 18s rDNA phylogenetic tree includes the unclassified nematode species (OR478781) from *G. macrorhynchus*, which clusters together with the Strongylidae family. The GenBank accession numbers are indicated before the species names and the numbers on the nodes represent the bootstrap confidence levels.

Our study aimed to understand the genetic makeup of a nematode parasite by examining a specific region of the 18s rDNA sequence using nested PCR. The DNA fragment we isolated and amplified measured 774 base pairs in size. To investigate the relationships and similarities between our sequence and others, we conducted a BLASTn analysis. The results showed that our sequence had matches with only a limited number of sequences in the GeneBank database. Among these matches, *Cyathostomum catinatum* exhibited the highest sequence identity at 83.95 with 98% of query coverage. The limited matches with other sequences can be attributed to the absence of specific sequences in the GeneBank database for Strongylidae species. Afterward, a multiple sequence alignment was performed using the MUSCLE algorithm in MEGAX including all the blastn sequences and additional 18s rDNA nucleotide sequences from the NCBI database. The final alignment size showed a total of 471bp (Supplementary Figure 2). Additionally, we constructed a phylogenetic tree (Figure 2) to illustrate the evolutionary relationships based on genetic sequences. Our isolated sequence formed a distinct branch compared to the *C. catinatum* sequence in the tree, although with low support from the bootstrap value, suggesting some uncertainty. This distinctive branch indicated significant genetic differentiation between our nematode parasite and *C. catinatum*. In addition, a pairwise distance matrix was created based on our isolated sequence (OR478781) with all the 18s rDNA gene sequences of *Cylicocyclus* spp. and *Cyathostomum* spp. from NCBI database for assessing genetic diversity and phylogenetic relationships among individuals or populations (Supplementary Tables 3, 4). The Supplementary Tables show that our isolated sequence has the closest relation with *C. ashworthi* (AJ223334) and *C. catinatum* (AJ223339). However, it is important to note that despite its unique position, our sequence clustered with other members of the Strongylidae family in the evolutionary tree. This suggests a more comprehensive genetic relationship among individuals from the same family, potentially indicating a shared ancestral lineage and certain genetic traits.

**Figure 3:**
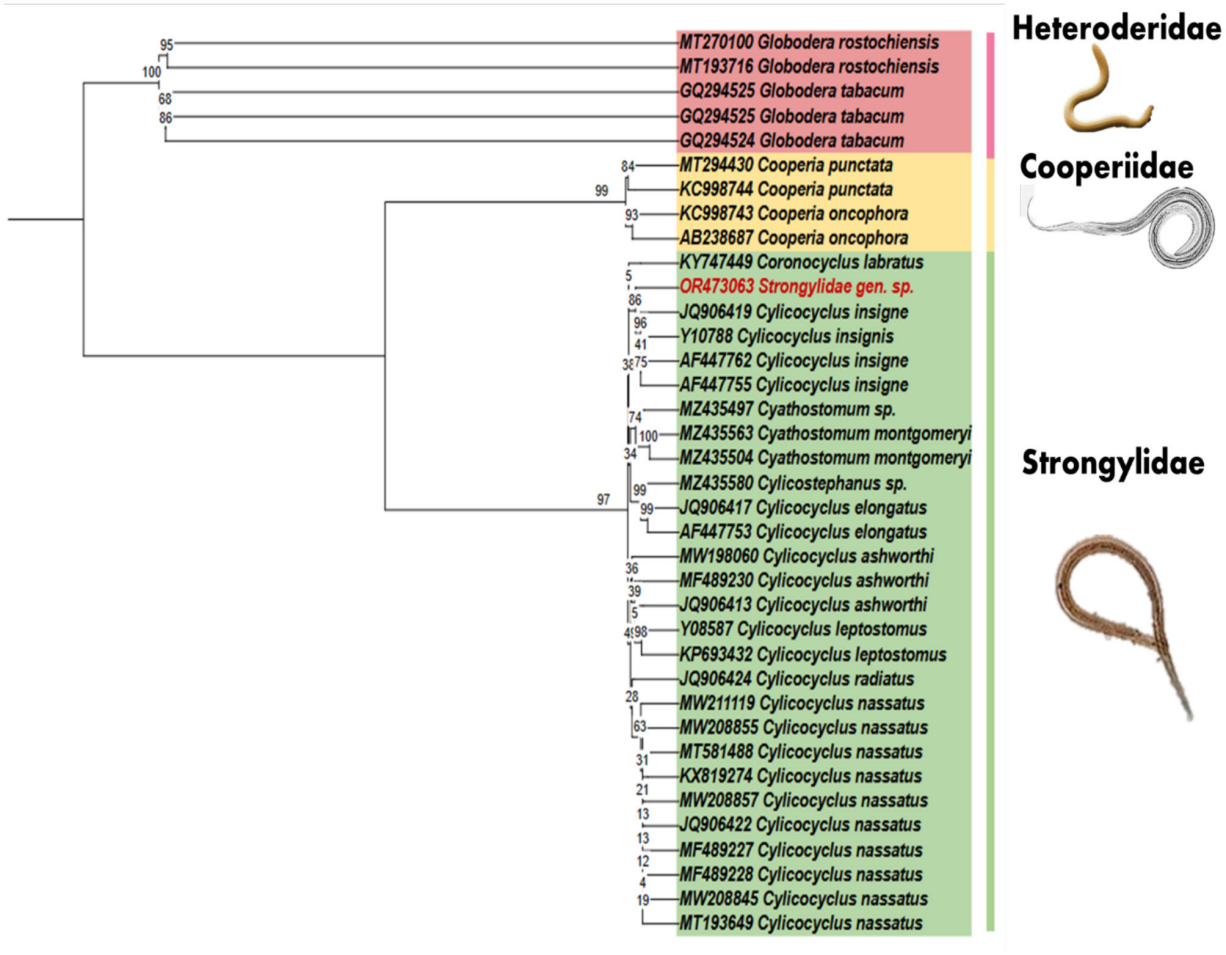
The maximum likelihood method is used to determine the evolutionary relationships between nematodes by analyzing ITS2 sequences. The bootstrap test, consisting of 1,000 replicates, shows the percentages of replicate trees in which the related taxa are grouped, and these values are displayed above the branches.

In addition, PCR amplification was used to generate a segment of the *ITS2* gene, and a 399-bp contig was formed after sequencing the PCR product. CD-HIT-est was applied against the ITS2 nemabiome dataset (https://www.nemabiome.ca/its2-database) containing 12,436 species for clustering analysis of the isolated sequence before performing BLASTn to identify the species in the genus and species level. The CD-HIT-est server analysis demonstrated that the isolated sequence is closely related to *Cylicocyclus nassatus* (96.29%) and *Cylicocyclus insigne* (93.75%) within the Strongylidae family. Moreover, the BLASTn result revealed a 96% sequence similarity to the *C. nassatus* species and 99.1% sequence similarity with *C. insigne*. However, the query coverage of *C. insigne* (83%) is lower than *C. nassatus*. Additionally, a multiple sequence alignment was conducted using the MUSCLE approach in MEGAX software, incorporating both the blastn sequences and an extra set of ITS nucleotide sequences obtained from the NCBI database. The ultimate alignment size exhibited an overall number of 245 bp (Supplementary Figure 3). Further, a phylogenetic tree based on our ITS2 sequence was constructed (Figure 3), which included various nematode families and multiple Strongylidae isolates from different geographic locations. The analysis of this tree consistently showed that our isolated sequence forms a distinct branch within the Strongylidae family, clustering closely with *C. insigne*. The pairwise distance matrix based on ITS2 sequences of *Cylicocyclus* spp. and *Cyathostomum* spp. (Supplementary Tables 5, 6, respectively) shows that the ITS2 sequence isolated from the dead whale has a closer relationship with *Cylicocyclus insigne* compared with *Cyathostomum* genus. Thus, based on the combined results of BLASTn, CD-HIT-est, and the ITS2 phylogenetic tree, it can be confidently concluded that our isolated sequence originates from the Strongylidae nematode family.

## 4 Discussion

The findings of this study offer significant contributions to our understanding of the taxonomy and phylogenetic position of the isolated nematode species. By employing microscopic observation and illustration, molecular techniques, and comparative analysis of all the 18s rDNA and ITS sequences of *Cylicocyclus* spp. and *Cyathostomum* spp., along with a cluster analysis of our isolated ITS2 sequence with nematode database, a comprehensive comprehension of the classification of the nematodes and its relationship within the Strongylidae family has been successfully attained.

The Strongylidae family, which can be further categorized into Strongylinae and Cyathostominae, encompasses a wide range of parasitic nematodes that reside in the gastrointestinal tracts of vertebrates. They are a type of nematode found in horses (13). They possess a well-developed buccal capsule, a mouth collar with two leaf crowns, and a copulatory bursa belonging to the strongyloid superfamily, commonly referred to as Superfamily Strongyloidea (13). The Strongylidae nematodes belonging to the superfamily Strongyloidea typically possess buccal capsules that are either large or medium-sized, shaped like globes or funnels. On the other hand, the buccal capsules of the Cyathostominae are usually cylindrical and range in size from tiny to medium (13). According to Lichtenfels et al. (13), male *Cylicocyclus* body length is normally 7.4–8.3 mm in length and female length ranges between 8.0–10.5 mm (26), which supports the finding of our study by light microscopic examination. However, during the collection of the parasite, some other key morphological features could be damaged. Furthermore, based on the morphological investigation, it can be inferred that the buccal cavity of the parasite obtained from the Pilot Whale was relatively small and cylindrical and body length indicates the parasite toward a Strongylidae family. Therefore, this finding indicates that the parasite is most likely a member of the compact and moderately sized Strongylidae family.

Moreover, the lacks of distinctive biogeographical patterns commonly observed in vertebrates and insects is not observed in nematodes. This could be due to insufficient data available for this group, or it could imply that nematodes possess unique biological and ecological traits as meio-organisms (32). Accurately estimating global nematode diversity is challenging due to the lack of a thorough assessment of nematode species diversity and phylogeographic patterns (11). It is postulated that many taxa found in the deep ocean have a wide distribution and may exhibit a cosmopolitan occurrence (33, 34), it is surprising that meiofaunal benthic creatures, which usually do not have planktonic larval stages, are still found to have a vast distribution in the deep ocean (35). Furthermore, the phylum Nematoda is found in various extreme environments, indicating its widespread presence. This is evident from the global distribution of numerous species of freshwater nematodes, as they are recorded across all continents (36).

Cetaceans have long been seen as having a close evolutionary relationship with primordial artiodactyls, as shown by the presence of an astragalus in some early whale fossils, which is a distinctive trait exclusive to the Artiodactyla order (37). Artiodactyla, including a wide range of extant mammals such as pigs, peccaries, deer, camels, llamas, alpacas, sheep, goats, cattle, and antelope, has been acknowledged for its diversity in terrestrial mammals for a considerable period (38). Numerous species of parasitic flatworms, namely those belonging to the taxonomic groups Cestoda and Trematoda, as well as roundworms of the Nematoda class, exhibit a lifecycle that involves residing inside the tissues of artiodactyl hosts (39). Among nematodes, Strongyloides nematode parasites, which are members of the Strongylidae family, are often seen in the small intestine of animals, especially ruminants and equines. These parasites possess a distinctive lifecycle that entails one or several generations of adult worms existing in a free-living state (40). Therefore, the findings and further analysis of this research provide compelling evidence that the Strongylidae parasite, obtained from the short-finned pilot whale, may have originated from a shared ancestor and potentially have a cosmopolitan distribution (15).

Traditional approaches focused on morphology have proven to be insufficient for identifying and exploring the diversity of nematodes. This is mainly because there are limited distinct morphological differences available between species (41). For several years, the internal transcribed spacer 2 (ITS2) rDNA locus has been extensively used for species identification in both free-living and parasitic nematodes (42). The molecular techniques used in this study, such as PCR amplification and sequencing, were effective in determining the genetic composition of the identified nematode sequence. While the 18s rDNA analysis did not provide differentiation for the isolated sequence, the application of the CD-HIT-est server against a comprehensive ITS2 nemabiome dataset proved to be useful (24). The practical clustering analysis revealed a strong correlation between the isolated sequence and the Strongylidae family, particularly with reference to *Cylicocyclus insigne*. This finding was reinforced by the BLASTn analysis, which indicated a considerable degree of sequence homology with the *C. insigne* species. The construction of the ITS2 phylogenetic tree encompassing various nematode families and Strongylidae isolates from diverse geographical locations yielded a significant revelation. The isolated sequence exhibited a distinct branch within the Strongylidae family, exhibiting a close clustering pattern alongside *C. insigne*. The consistent clustering further strengthens the reliability of the findings, reinforcing the inference that the isolated sequence is affiliated with the Strongylidae nematode family. Additionally, this clustering pattern suggests the wide distribution of the genus *Cylicocyclus*.

## 5 Conclusion

This study uses diverse molecular sequence analysis to identify and classify an isolated nematode species from a short-finned Pilot Whale within the Strongylidae family. Traditional morphology-based methods were not sufficient in this study due to the lack of number of parasites, so molecular analyses of the 18s rDNA and ITS2 rDNA were conducted to identify the parasite more convincingly. The results confirm a strong connection to Strongylidae, particularly the *Cylicocyclus* genus, suggesting widespread distribution within this genus. This study highlights the importance of using molecular tools and interdisciplinary approaches to understand the complexities of nematode diversity, evolution, and ecological interactions in marine ecosystems. By comprehensive molecular sequence analysis, this research enhances our knowledge of nematode dynamics and their ecological significance in marine habitats. The findings of Strongylidae parasite in the marine mammal from this study describe the dynamic nature of the equine *Cyclicocyclus* genus and emphasize the necessity of taking a multidimensional perspective to fully comprehend the complexities of nematode biology and their broader implications in marine environments.

## Data availability statement

The datasets presented in this study can be found in online repositories. The names of the repository/repositories and accession number(s) can be found below: GenBank, OR478781.

## Ethics statement

Ethical approval was not required for the study involving animals in accordance with the local legislation and institutional requirements because this parasite was collected from a necropsy animal.

## Author contributions

WJ: Investigation, Methodology, Writing – original draft. SI: Investigation, Methodology, Writing – original draft. AT: Investigation, Methodology, Writing – original draft. PS: Investigation, Methodology, Writing – original draft. AJ: Investigation, Methodology, Writing – original draft. KT: Investigation, Methodology, Writing – original draft. RP: Investigation, Methodology, Writing – original draft. PT: Funding acquisition, Methodology, Supervision, Writing – review & editing.
