# Supplementary material for: First report of Strongylidae nematode from pilot whale (Globicephala macrorhynchus) by molecular analysis reveals the cosmopolitan distribution of the taxon
Source: Front Vet Sci. 2023 Dec 7;10:1313783. doi: 10.3389/fvets.2023.1313783 (PMC10755461; doi:10.3389/fvets.2023.1313783)
Supplement: Supplementary file 1 [file Data_Sheet_1.docx]

Supplementary Material

## Supplementary Figures
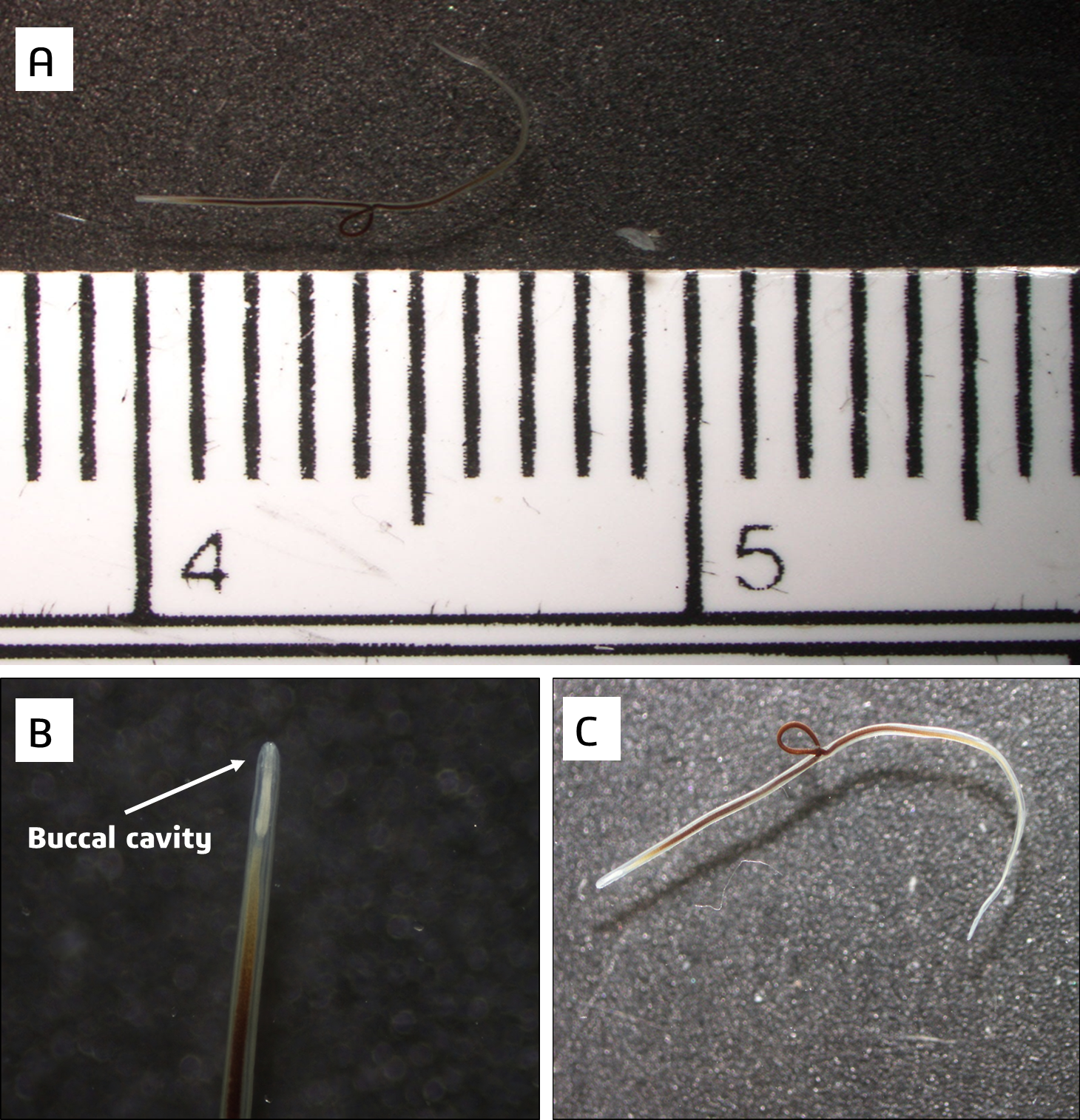
Supplementary Figure 1. (A) Full body length of the isolated parasite. (B) A close-up view reveals the buccal cavity of a specific nematode species, indicated by an arrow. (C) The entire body of the nematode species is visible, with the intestine also visible to the naked eye.


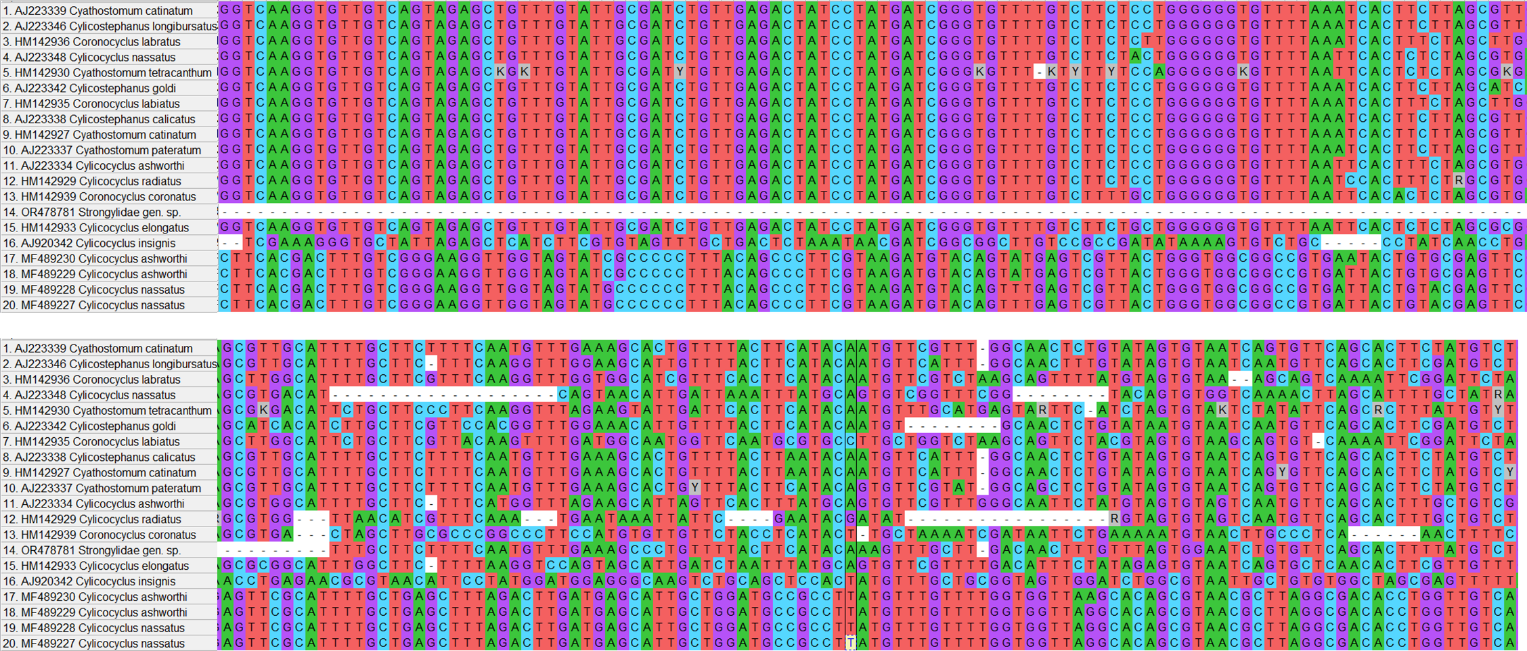


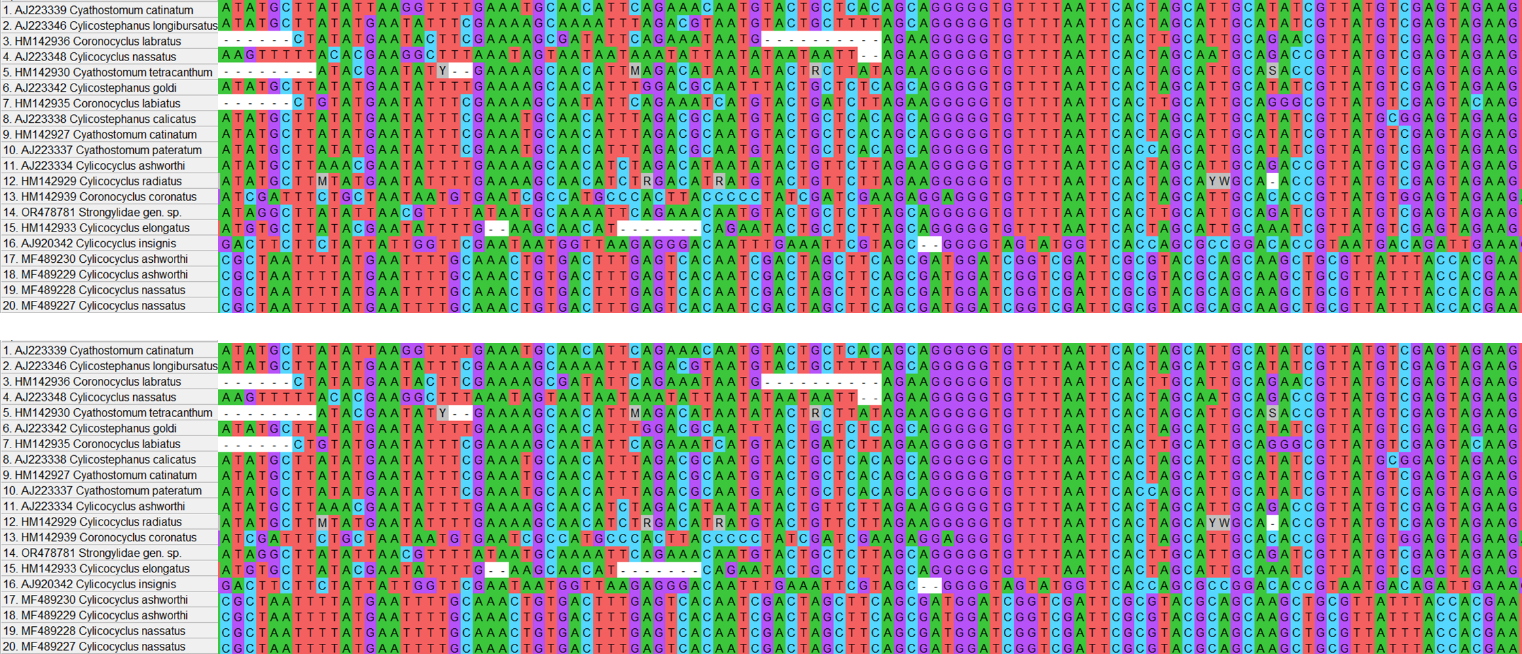

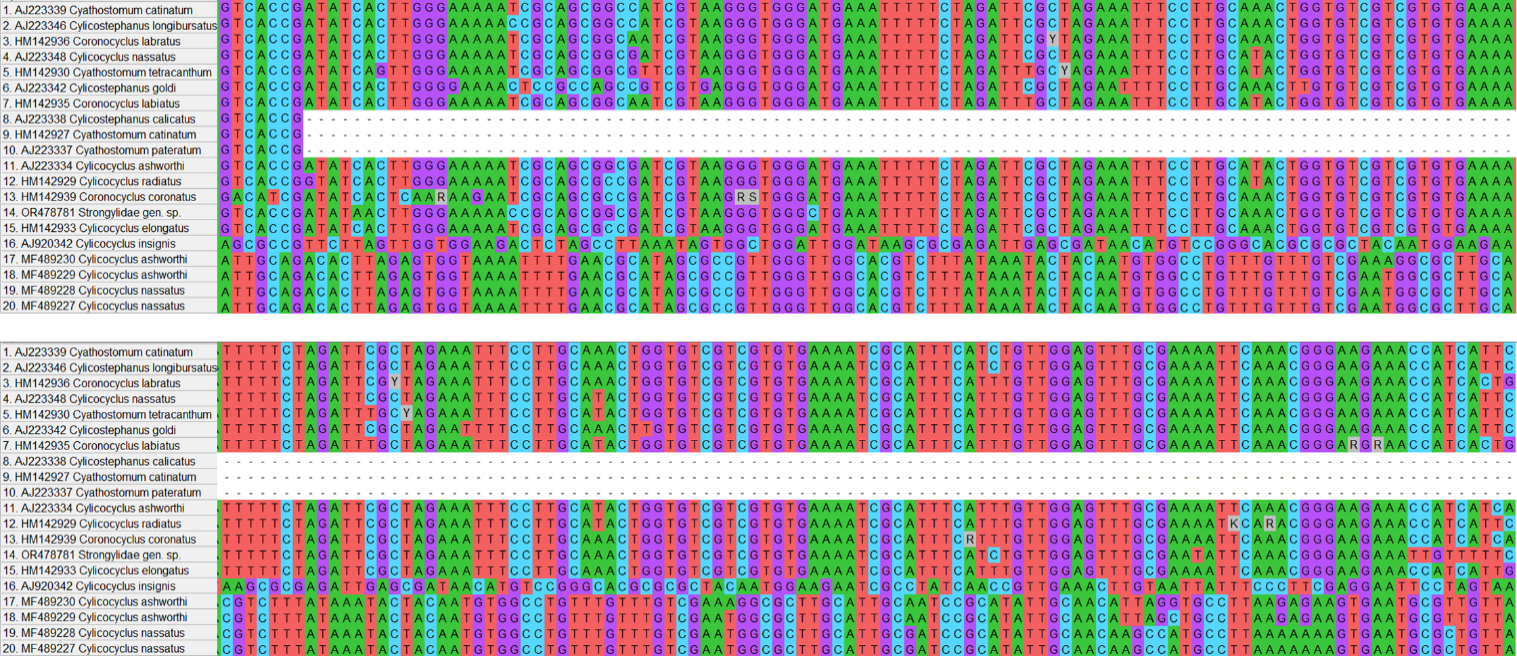
**Supplementary Figure 2**. Multiple sequence alignment based on Strongylidae 18s rDNA sequences from blastn and NCBI database

**
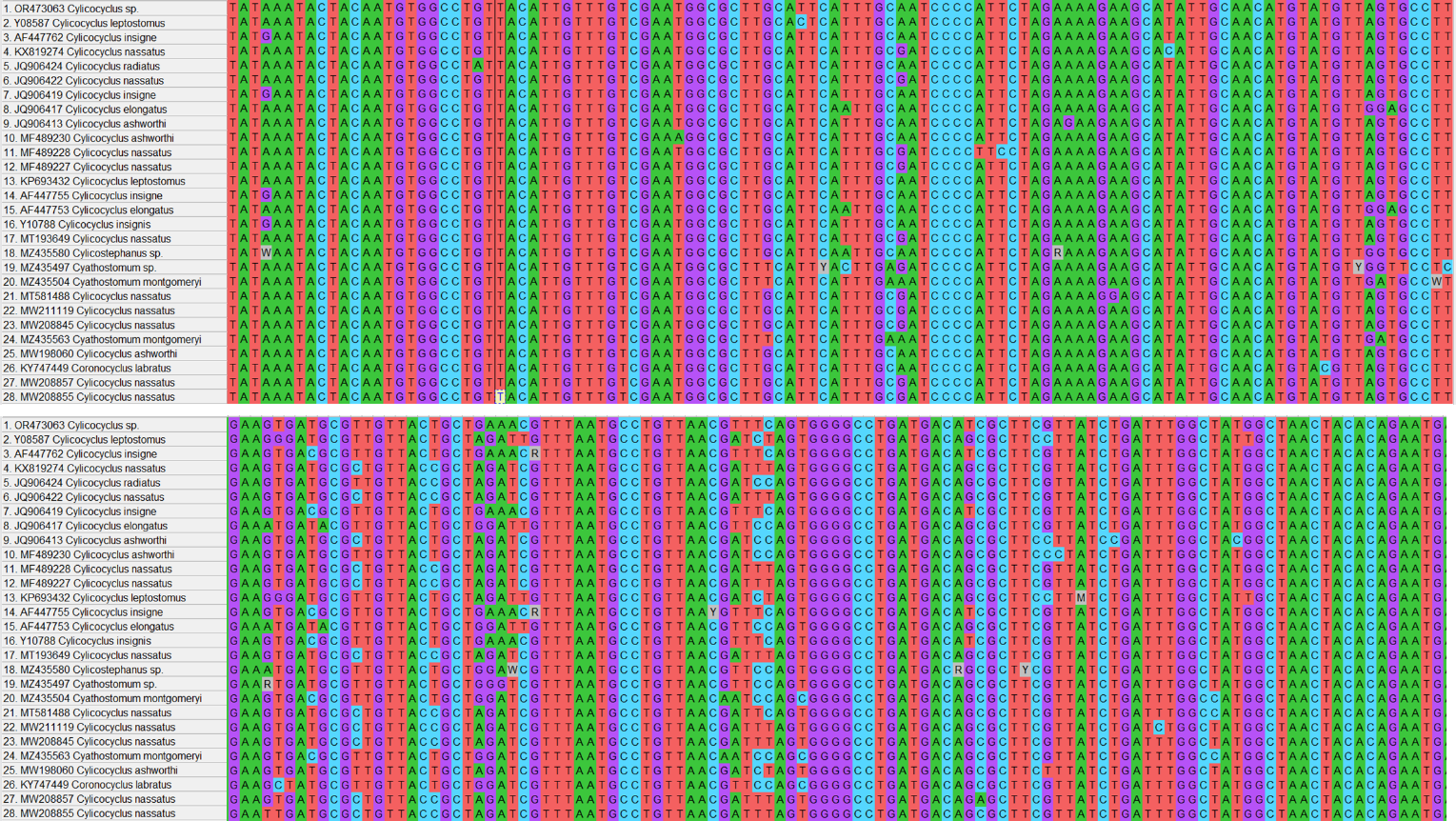
**

**
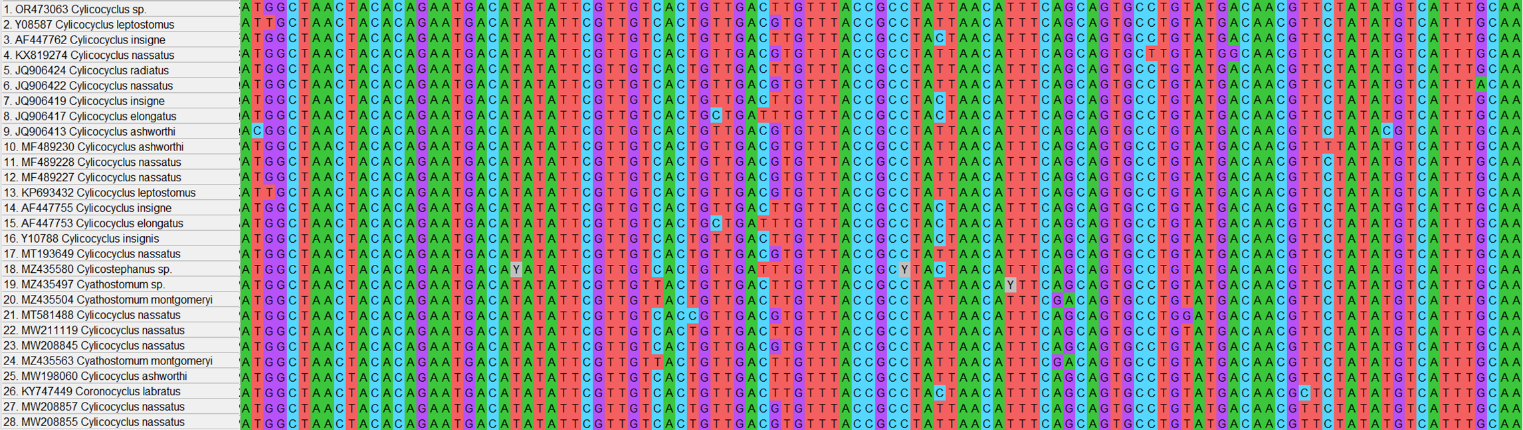
**

**Supplementary Figure 3**. Multiple sequence alignment based on Strongylidae ITS2 sequences from blastn and NCBI database

**2. Supplementary Tables**

**Supplementary Table 1.** BLASTn result from the 18s rDNA gene sequence from the isolated nematode parasite

| **Scientific Name** | **Max Score** | **Total Score** | **Query Cover** | **E value** | **Per. ident** | **Acc. Len** | **Accession** |
| --- | --- | --- | --- | --- | --- | --- | --- |
| *Cyathostomum catinatum* | 682 | 682 | 98% | 0 | 83.95 | 1458 | [AJ223339.1](https://www.ncbi.nlm.nih.gov/nucleotide/AJ223339.1?report=genbank&log$=nucltop&blast_rank=1&RID=EG4KZJ27013) |
| *Cylicostephanus longibursatus* | 499 | 499 | 98% | 4.00E-136 | 79.79 | 1444 | [AJ223346.1](https://www.ncbi.nlm.nih.gov/nucleotide/AJ223346.1?report=genbank&log$=nucltop&blast_rank=2&RID=EG4KZJ27013) |
| *Coronocyclus labratus* | 399 | 399 | 35% | 4.00E-106 | 93.07 | 1449 | [HM142936.1](https://www.ncbi.nlm.nih.gov/nucleotide/HM142936.1?report=genbank&log$=nucltop&blast_rank=3&RID=EG4KZJ27013) |
| *Cylicocyclus nassatus* | 385 | 385 | 37% | 1.00E-101 | 90.54 | 1466 | [AJ223348.1](https://www.ncbi.nlm.nih.gov/nucleotide/AJ223348.1?report=genbank&log$=nucltop&blast_rank=4&RID=EG4KZJ27013) |
| *Cyathostomum tetracanthum* | 374 | 374 | 37% | 2.00E-98 | 89.93 | 1106 | [HM142930.1](https://www.ncbi.nlm.nih.gov/nucleotide/HM142930.1?report=genbank&log$=nucltop&blast_rank=5&RID=EG4KZJ27013) |
| *Cylicostephanus goldi* | 374 | 374 | 80% | 2.00E-98 | 78.79 | 1451 | [AJ223342.1](https://www.ncbi.nlm.nih.gov/nucleotide/AJ223342.1?report=genbank&log$=nucltop&blast_rank=6&RID=EG4KZJ27013) |
| *Coronocyclus labiatus* | 370 | 370 | 33% | 3.00E-97 | 92.37 | 1839 | [HM142935.1](https://www.ncbi.nlm.nih.gov/nucleotide/HM142935.1?report=genbank&log$=nucltop&blast_rank=7&RID=EG4KZJ27013) |
| *Cylicostephanus calicatus* | 368 | 368 | 66% | 1.00E-96 | 80.51 | 694 | [AJ223338.1](https://www.ncbi.nlm.nih.gov/nucleotide/AJ223338.1?report=genbank&log$=nucltop&blast_rank=8&RID=EG4KZJ27013) |
| *Cyathostomum catinatum* | 357 | 357 | 66% | 2.00E-93 | 79.73 | 694 | [HM142927.1](https://www.ncbi.nlm.nih.gov/nucleotide/HM142927.1?report=genbank&log$=nucltop&blast_rank=9&RID=EG4KZJ27013) |
| *Cyathostomum pateratum* | 357 | 357 | 65% | 2.00E-93 | 80.2 | 693 | [AJ223337.1](https://www.ncbi.nlm.nih.gov/nucleotide/AJ223337.1?report=genbank&log$=nucltop&blast_rank=10&RID=EG4KZJ27013) |

**Supplementary Table 2.** BLASTn result from the ITS2 gene sequence from the isolated nematode parasite

| **Scientific Name** | **Max Score** | **Total Score** | **Query Cover** | **E value** | **Per. ident** | **Acc. Len** | **Accession** |
| --- | --- | --- | --- | --- | --- | --- | --- |
| *Cylicocyclus nassatus* | 634 | 634 | 97% | 5.00E-177 | 96.14 | 514 | [MT193649.1](https://www.ncbi.nlm.nih.gov/nucleotide/MT193649.1?report=genbank&log$=nucltop&blast_rank=1&RID=EG5B0WN8013) |
| *Cylicostephanus* sp*.* | 632 | 632 | 99% | 2.00E-176 | 94.72 | 412 | [MZ435580.1](https://www.ncbi.nlm.nih.gov/nucleotide/MZ435580.1?report=genbank&log$=nucltop&blast_rank=2&RID=EG5B0WN8013) |
| *Cyathostomum montgomeryi* | 623 | 623 | 100% | 1.00E-173 | 94.99 | 411 | [MZ435572.1](https://www.ncbi.nlm.nih.gov/nucleotide/MZ435572.1?report=genbank&log$=nucltop&blast_rank=3&RID=EG5B0WN8013) |
| *Cyathostomum* sp*.* | 614 | 614 | 97% | 6.00E-171 | 94.86 | 397 | [MZ435497.1](https://www.ncbi.nlm.nih.gov/nucleotide/MZ435497.1?report=genbank&log$=nucltop&blast_rank=4&RID=EG5B0WN8013) |
| *Cyathostomum montgomeryi* | 608 | 608 | 100% | 3.00E-169 | 94.24 | 410 | [MZ435504.1](https://www.ncbi.nlm.nih.gov/nucleotide/MZ435504.1?report=genbank&log$=nucltop&blast_rank=5&RID=EG5B0WN8013) |
| *Cylicocyclus nassatus* | 604 | 604 | 96% | 4.00E-168 | 95.06 | 893 | [MT581488.1](https://www.ncbi.nlm.nih.gov/nucleotide/MT581488.1?report=genbank&log$=nucltop&blast_rank=6&RID=EG5B0WN8013) |
| *Cyathostomum montgomeryi* | 603 | 603 | 97% | 1.00E-167 | 94.62 | 395 | [MZ435583.1](https://www.ncbi.nlm.nih.gov/nucleotide/MZ435583.1?report=genbank&log$=nucltop&blast_rank=7&RID=EG5B0WN8013) |
| *Cyathostomum montgomeryi* | 603 | 603 | 100% | 1.00E-167 | 93.98 | 410 | [MZ435553.1](https://www.ncbi.nlm.nih.gov/nucleotide/MZ435553.1?report=genbank&log$=nucltop&blast_rank=8&RID=EG5B0WN8013) |
| *Cyathostomum montgomeryi* | 603 | 603 | 100% | 1.00E-167 | 93.98 | 411 | [MZ435551.1](https://www.ncbi.nlm.nih.gov/nucleotide/MZ435551.1?report=genbank&log$=nucltop&blast_rank=9&RID=EG5B0WN8013) |
| *Cylicocyclus insigne* | 603 | 603 | 83% | 1.00E-167 | 99.1 | 843 | [JQ906419.1](https://www.ncbi.nlm.nih.gov/nucleotide/JQ906419.1?report=genbank&log$=nucltop&blast_rank=10&RID=EG5B0WN8013) |

Supplementary Table 3. Pairwise distance matrix between our isolated sequence and available 18s rDNA sequence of *Cylicocyclus* spp. in GenBank

|  | 1 | 2 | 3 | 4 | 5 | 6 | 7 | 8 | 9 | 10 |
| --- | --- | --- | --- | --- | --- | --- | --- | --- | --- | --- |
| 1. OR478781 | * |  |  |  |  |  |  |  |  |  |
| 2. AJ920342 | 0.88 | * |  |  |  |  |  |  |  |  |
| 3. MF489230 | 0.75 | 1.37 | * |  |  |  |  |  |  |  |
| 4. MF489229 | 0.76 | 1.39 | 0.01 | * |  |  |  |  |  |  |
| 5. MF489228 | 0.75 | 1.41 | 0.04 | 0.03 | * |  |  |  |  |  |
| 6. MF489227 | 0.74 | 1.40 | 0.04 | 0.03 | 0.00 | * |  |  |  |  |
| 7. HM142933 | 0.29 | 0.80 | 0.85 | 0.85 | 0.87 | 0.86 | * |  |  |  |
| 8. HM142929 | 0.30 | 0.80 | 0.72 | 0.73 | 0.75 | 0.74 | 0.20 | * |  |  |
| 9. AJ223348 | 0.37 | 0.81 | 0.83 | 0.85 | 0.85 | 0.84 | 0.26 | 0.25 | * |  |
| 10. AJ223334 | 0.27 | 0.75 | 0.80 | 0.81 | 0.83 | 0.82 | 0.17 | 0.10 | 0.22 | * |

**Supplementary Table 4**. Pairwise distance matrix between our isolated sequence and available 18s rDNA sequence of *Cyathostomum* spp. in GenBank

|  | 1 | 2 | 3 | 4 | 5 |
| --- | --- | --- | --- | --- | --- |
| 1. OR478781 | * |  |  |  |  |
| 2. AJ223339 | 0.12 | * |  |  |  |
| 3. AJ223727 | 0.23 | 0.15 | * |  |  |
| 4. HM142930 | 0.21 | 0.13 | 0.14 | * |  |
| 5. MF489231 | 0.80 | 0.82 | 0.68 | 0.83 | * |

**Supplementary Table 5**. Pairwise distance matrix between our isolated sequence and available ITS2 sequence of *Cylicocyclus* spp. in GenBank

|  | | 1 | 2 | | 3 | | 4 | | 5 | | 6 | | 7 | | 8 | | 9 | | 10 | | 11 | | 12 | | 13 | | 14 | | 15 | | 16 | | 17 | | 18 | | 19 | | 20 | | 21 | |
| --- | --- | --- | --- | --- | --- | --- | --- | --- | --- | --- | --- | --- | --- | --- | --- | --- | --- | --- | --- | --- | --- | --- | --- | --- | --- | --- | --- | --- | --- | --- | --- | --- | --- | --- | --- | --- | --- | --- | --- | --- | --- | --- |
| 1. OR473063 | * | | |  | |  | |  | |  | |  | |  | |  | |  | |  | |  | |  | |  | |  | |  | |  | |  | |  | |  | |  | |  |
| 2. Y08587 | | 0.05 | * | |  | |  | |  | |  | |  | |  | |  | |  | |  | |  | |  | |  | |  | |  | |  | |  | |  | |  | |  | |
| 3. AJ004836 | | 0.18 | 0.18 | | * | |  | |  | |  | |  | |  | |  | |  | |  | |  | |  | |  | |  | |  | |  | |  | |  | |  | |  | |
| 4. AJ004835 | | 0.06 | 0.07 | | 0.20 | | * | |  | |  | |  | |  | |  | |  | |  | |  | |  | |  | |  | |  | |  | |  | |  | |  | |  | |
| 5. AF447762 | | 0.01 | 0.06 | | 0.18 | | 0.07 | | * | |  | |  | |  | |  | |  | |  | |  | |  | |  | |  | |  | |  | |  | |  | |  | |  | |
| 6. KX819274 | | 0.06 | 0.04 | | 0.20 | | 0.07 | | 0.07 | | * | |  | |  | |  | |  | |  | |  | |  | |  | |  | |  | |  | |  | |  | |  | |  | |
| 7. JQ906424 | | 0.04 | 0.03 | | 0.18 | | 0.05 | | 0.05 | | 0.02 | | * | |  | |  | |  | |  | |  | |  | |  | |  | |  | |  | |  | |  | |  | |  | |
| 8. JQ906422 | | 0.05 | 0.04 | | 0.19 | | 0.07 | | 0.06 | | 0.00 | | 0.01 | | * | |  | |  | |  | |  | |  | |  | |  | |  | |  | |  | |  | |  | |  | |
| 9. JQ906419 | | 0.01 | 0.06 | | 0.18 | | 0.07 | | 0.00 | | 0.03 | | 0.02 | | 0.03 | | * | |  | |  | |  | |  | |  | |  | |  | |  | |  | |  | |  | |  | |
| 10. JQ906417 | | 0.06 | 0.06 | | 0.18 | | 0.07 | | 0.06 | | 0.04 | | 0.03 | | 0.04 | | 0.03 | | * | |  | |  | |  | |  | |  | |  | |  | |  | |  | |  | |  | |
| 11. JQ906416 | | 0.03 | 0.07 | | 0.19 | | 0.08 | | 0.04 | | 0.04 | | 0.03 | | 0.04 | | 0.02 | | 0.03 | | * | |  | |  | |  | |  | |  | |  | |  | |  | |  | |  | |
| 12. JQ906413 | | 0.05 | 0.03 | | 0.19 | | 0.06 | | 0.06 | | 0.02 | | 0.01 | | 0.02 | | 0.03 | | 0.03 | | 0.03 | | * | |  | |  | |  | |  | |  | |  | |  | |  | |  | |
| 13. JQ906411 | | 0.10 | 0.11 | | 0.23 | | 0.12 | | 0.10 | | 0.05 | | 0.04 | | 0.05 | | 0.05 | | 0.05 | | 0.05 | | 0.05 | | * | |  | |  | |  | |  | |  | |  | |  | |  | |
| 14MF489230 | | 0.04 | 0.03 | | 0.19 | | 0.06 | | 0.05 | | 0.02 | | 0.01 | | 0.02 | | 0.03 | | 0.04 | | 0.03 | | 0.01 | | 0.05 | | * | |  | |  | |  | |  | |  | |  | |  | |
| 15. MF489228 | | 0.05 | 0.04 | | 0.19 | | 0.07 | | 0.06 | | 0.01 | | 0.02 | | 0.00 | | 0.03 | | 0.04 | | 0.04 | | 0.02 | | 0.05 | | 0.02 | | * | |  | |  | |  | |  | |  | |  | |
| 16. MF489227 | | 0.05 | 0.03 | | 0.19 | | 0.07 | | 0.06 | | 0.00 | | 0.01 | | 0.00 | | 0.03 | | 0.03 | | 0.03 | | 0.02 | | 0.05 | | 0.02 | | 0.00 | | * | |  | |  | |  | |  | |  | |
| 17. KP693432 | | 0.04 | 0.00 | | 0.18 | | 0.07 | | 0.05 | | 0.02 | | 0.01 | | 0.02 | | 0.03 | | 0.03 | | 0.03 | | 0.01 | | 0.05 | | 0.01 | | 0.02 | | 0.02 | | * | |  | |  | |  | |  | |
| 18. KP693431 | | 0.18 | 0.18 | | 0.00 | | 0.20 | | 0.18 | | 0.14 | | 0.12 | | 0.13 | | 0.13 | | 0.13 | | 0.13 | | 0.13 | | 0.15 | | 0.13 | | 0.13 | | 0.13 | | 0.12 | | * | |  | |  | |  | |
| 19. AF447755 | | 0.01 | 0.06 | | 0.18 | | 0.07 | | 0.00 | | 0.07 | | 0.05 | | 0.06 | | 0.00 | | 0.06 | | 0.04 | | 0.06 | | 0.10 | | 0.05 | | 0.06 | | 0.06 | | 0.05 | | 0.18 | | * | |  | |  | |
| 20.AF447753 | | 0.06 | 0.06 | | 0.18 | | 0.07 | | 0.06 | | 0.07 | | 0.05 | | 0.07 | | 0.06 | | 0.00 | | 0.07 | | 0.07 | | 0.11 | | 0.06 | | 0.07 | | 0.06 | | 0.06 | | 0.18 | | 0.06 | | * | |  | |
| 21. Y10788 | | 0.01 | 0.06 | | 0.18 | | 0.07 | | 0.00 | | 0.07 | | 0.05 | | 0.06 | | 0.00 | | 0.06 | | 0.04 | | 0.06 | | 0.10 | | 0.05 | | 0.06 | | 0.06 | | 0.05 | | 0.18 | | 0.00 | | 0.06 | | * | |

**Supplementary Table 6**. Pairwise distance matrix between our isolated sequence and available ITS sequence of *Cyathostomum* spp. in GenBank

|  | 1 | 2 | 3 | 4 | 5 | 6 | 7 | 8 | 9 | 10 | 11 | 12 | 13 | 14 | 15 | 16 | 17 | 18 |
| --- | --- | --- | --- | --- | --- | --- | --- | --- | --- | --- | --- | --- | --- | --- | --- | --- | --- | --- |
| 1. OR473063 | * |  |  |  |  |  |  |  |  |  |  |  |  |  |  |  |  |  |
| 2. MG727532 | 0.03 | * |  |  |  |  |  |  |  |  |  |  |  |  |  |  |  |  |
| 3. AF263496 | 0.03 | 0.01 | * |  |  |  |  |  |  |  |  |  |  |  |  |  |  |  |
| 4. OK235480 | 0.03 | 0.05 | 0.04 | * |  |  |  |  |  |  |  |  |  |  |  |  |  |  |
| 5. OK235479 | 0.04 | 0.06 | 0.06 | 0.04 | * |  |  |  |  |  |  |  |  |  |  |  |  |  |
| 6. OK235474 | 0.03 | 0.04 | 0.04 | 0.02 | 0.04 | * |  |  |  |  |  |  |  |  |  |  |  |  |
| 7. OK235471 | 0.04 | 0.06 | 0.06 | 0.02 | 0.05 | 0.01 | * |  |  |  |  |  |  |  |  |  |  |  |
| 8. OK235469 | 0.03 | 0.04 | 0.04 | 0.02 | 0.04 | 0.01 | 0.00 | * |  |  |  |  |  |  |  |  |  |  |
| 10. OK235467 | 0.05 | 0.07 | 0.06 | 0.03 | 0.07 | 0.04 | 0.03 | 0.03 | * |  |  |  |  |  |  |  |  |  |
| 11. MF489231 | 0.04 | 0.03 | 0.05 | 0.03 | 0.06 | 0.02 | 0.04 | 0.02 | 0.07 | * |  |  |  |  |  |  |  |  |
| 12. KY495603 | 0.03 | 0.01 | 0.01 | 0.03 | 0.05 | 0.03 | 0.05 | 0.03 | 0.05 | 0.03 | * |  |  |  |  |  |  |  |
| 13. KF850628 | 0.03 | 0.00 | 0.01 | 0.04 | 0.06 | 0.04 | 0.06 | 0.04 | 0.06 | 0.03 | 0.01 | * |  |  |  |  |  |  |
| 14. KF850626 | 0.03 | 0.01 | 0.01 | 0.03 | 0.05 | 0.03 | 0.05 | 0.03 | 0.05 | 0.03 | 0.00 | 0.01 | * |  |  |  |  |  |
| 15. AJ004838 | 0.03 | 0.03 | 0.03 | 0.01 | 0.06 | 0.04 | 0.03 | 0.03 | 0.05 | 0.04 | 0.03 | 0.03 | 0.03 | * |  |  |  |  |
| 16. AJ004837 | 0.02 | 0.02 | 0.02 | 0.03 | 0.04 | 0.03 | 0.04 | 0.03 | 0.05 | 0.04 | 0.02 | 0.02 | 0.02 | 0.03 | * |  |  |  |
| 17. Y08619 | 0.03 | 0.01 | 0.00 | 0.05 | 0.06 | 0.04 | 0.06 | 0.04 | 0.07 | 0.05 | 0.01 | 0.00 | 0.01 | 0.03 | 0.02 | * |  |  |
| 18. Y08583 | 0.03 | 0.01 | 0.00 | 0.05 | 0.06 | 0.04 | 0.06 | 0.04 | 0.07 | 0.05 | 0.01 | 0.00 | 0.01 | 0.03 | 0.02 | 0.00 | * |  |
| 19. Y08584 | 0.03 | 0.04 | 0.03 | 0.03 | 0.06 | 0.04 | 0.05 | 0.05 | 0.07 | 0.05 | 0.03 | 0.04 | 0.03 | 0.03 | 0.03 | 0.04 | 0.04 | * |
